# A Prompt-Guided and Quality-Aware Robust Text–Audio Intent Recognition Framework for Elderly Care

**DOI:** 10.3390/s26165233

**Published:** 2026-08-18

**Authors:** Zhimin Wei, Shuhao Tian, Yanzhen Wang, Yao Wang, Xiaolong Zhou, Jianyong Li

**Affiliations:** College of Mechanical Engineering, Beihua University, Jilin 132021, China

**Keywords:** multimodal intent recognition, elderly-care interaction, acoustic degradation, quality-aware fusion

## Abstract

In natural language understanding, intent recognition plays a central role in human–computer interaction. However, in elderly-care scenarios, acoustic signals are often affected by atypical speech patterns, slower speaking rates, and environmental noise, making audio information less reliable and reducing the effectiveness of conventional text–audio fusion methods. To address this problem, we propose a prompt-guided and quality-aware text–audio intent recognition framework. Specifically, a *χ*^2^-based intent prototype soft prompt is introduced to enhance the semantic representation of text. Then, a residual-free text-guided cross-attention module is designed to refine degraded acoustic features using textual semantics as reliable guidance. In addition, a dynamic fusion gate is developed to adjust the contributions of text and audio based on modality reliability and intent-related information. Experiments on the MIntRec dataset with simulated acoustic degradation show that the proposed model achieves 60.90% accuracy, 60.80% weighted F1, and 57.90% macro-F1, outperforming several competitive baselines. These results indicate that the proposed framework can improve the robustness of intent recognition under challenging acoustic conditions in elderly-oriented interaction scenarios.

## 1. Introduction

According to recent census data, population aging in China has reached a level comparable to the global average; consequently, ensuring the quality of life for elderly individuals—especially those with disabilities or living alone—has become an increasingly urgent societal concern. Within this context, mobile care robots have gradually emerged as important tools for improving the quality of life of older adults [1,2]. However, owing to the general decline in physiological functions among elderly users, their speech may exhibit unclear articulation or semantic ambiguity, making it challenging for traditional human–computer interaction (HCI) systems to accurately interpret their actual needs. As a central task in natural language understanding, intent recognition focuses on enabling machines to accurately infer users’ underlying intentions [3,4]. Multimodal intent recognition technology, by jointly analyzing users’ textual semantics and non-verbal features such as acoustic characteristics, can effectively address the limitations of unimodal methods, thereby providing essential support for natural and precise age-friendly interaction systems [5].

In recent years, deep learning-based multimodal language understanding has advanced considerably. Existing approaches predominantly rely on pre-trained language models (such as BERT) or Transformer-based architectures for cross-modal feature alignment and fusion [6]. With respect to specific fusion strategies, Li et al. proposed a recognition framework based on Modality Saliency Enhancement (L-MoSE) and Text-Guided Feature Modulation (TGMF) [7]. This method effectively highlights key semantic regions and improves the expressive power of multimodal features. However, its saliency evaluation relies heavily on the internal mapping of degraded signals, and the fusion phase lacks explicit consideration of real-time modality quality. To improve semantic representation for non-textual modalities, Li et al. developed a text-centered adaptive fusion gating and cross-modal attention mechanism [8]. This mechanism utilizes clean text as a guide to selectively filter redundant background noise, thereby enhancing cross-modal interaction quality. Nevertheless, the residual connections maintained in its architecture are prone to causing direct propagation of contaminated features under extreme noise conditions. In addition, Arevalo et al. introduced Gated Multimodal Units (GMU) to learn intermediate representations from multi-source data through internal multiplicative gating [9], effectively increasing the model’s adaptability to heterogeneous data sources. Meanwhile, Hu et al. proposed Dynamic Attention-based Fusion (DAF) along with a multi-view contrastive learning framework [10], which exhibited strong capability in bridging semantic gaps across modalities and optimizing cross-modal interaction weights.

Although the aforementioned approaches perform well under typical general-purpose conditions, the quality of multimodal data is often suboptimal when such systems are directly applied to real-world home care environments for the elderly. On the one hand, elderly users generally experience a decline in physiological functions, often leading to communication challenges such as slow speech, slurred pronunciation, and pronounced accents. On the other hand, home environments typically contain substantial levels of uncontrollable background noise and room reverberation. These real-world factors can cause severe degradation of acoustic signals. When a particular modality is severely contaminated, traditional static feature fusion techniques (such as feature concatenation or fixed-weight summation) not only fail to adequately isolate interference but also induce a “noise propagation” effect, thereby degrading the originally clean textual semantics [11]. Furthermore, even when using conventional gated fusion networks, severely degraded feature inputs can lead to a “routing collapse” problem characterized by over-reliance on a single modality, resulting in a significant decline in the model’s overall robustness in complex interactive scenarios [12].

To overcome these limitations, this paper presents a robust multimodal intent recognition approach tailored for degraded environments. To improve discrimination among similar intents in everyday conversations, this paper constructs an IP-SPT module based on the *χ*^2^ statistic for the text modality, which effectively guides the language model to capture intricate intent features [13]. Second, AudioAug is used to simulate noisy acoustic conditions, and a Text-Guided Cross-Attention mechanism [8] is developed to mitigate degradation of the resulting acoustic features. This mechanism leverages clean textual semantics as query representations to perform deep cross-modal refinement of degraded audio features, effectively limiting the propagation of environmental noise into the fusion process. Finally, a Quality-Type Dynamic Fusion mechanism (RTDF) is introduced to achieve adaptive weight allocation by evaluating the reliability of modalities and the current intent type in real time [14]; building on this, supervised contrastive learning [15] is introduced, using clean text features as anchors to constrain the multimodal fusion space, thereby mitigating gating collapse and feature drift under extreme degradation conditions.

Unlike existing multimodal intent recognition methods, the novelty of the proposed framework does not arise from simply combining prompt learning, cross-modal attention, dynamic fusion, and contrastive learning, but from redesigning these components specifically for unreliable acoustic information. Specifically, unlike general prompt tuning, the proposed IP-SPT uses the *χ*^2^ statistic to identify prototype words for individual intents and maps them to continuous prompts, thereby introducing explicit category-discriminative prior information into fine-grained intent recognition. Compared to existing text-guided cross-modal attention methods [8], the proposed cross-attention module removes the direct residual path of degraded acoustic features, preventing corrupted audio information from bypassing text-guided filtering. Furthermore, unlike traditional dynamic fusion methods that primarily learn modal interaction weights, RTDF jointly considers modality-quality representations and intent-related information, and adaptively adjusts each modality’s contribution according to the current input conditions. Finally, supervised contrastive learning is used as a representation constraint, with text features serving as semantic anchors rather than as an independent module. These design elements collectively form a unified, robustness-oriented framework designed to reduce noise propagation, unreliable modality fusion, and fine-grained intent ambiguity under acoustically degraded conditions.

The main contributions of this study are summarized as follows. First, for fine-grained intent recognition, we propose an Intent-Prototype Soft Prompting (IP-SPT) mechanism. Unlike conventional soft-prompt learning, IP-SPT utilizes *χ*^2^-based, category-discriminative prototype words to construct task-specific semantic priors, thereby improving discrimination among easily confused intents. Second, to address corrupted acoustic inputs, we design a residual-free text-guided cross-modal attention mechanism. Unlike traditional text-guided attention architectures, this mechanism removes the direct residual path of corrupted acoustic features, requiring acoustic information to undergo text-guided refinement before fusion, thereby reducing noise propagation. Third, we introduce a quality-aware dynamic fusion strategy that adaptively adjusts the weights of text and audio based on modal reliability and intent-relevant information, instead of relying on fixed or purely interaction-based fusion weights. Combined with supervised contrastive learning based on text anchors, these components collectively form a unified, robustness-oriented framework that maintains discriminative intent representations as acoustic reliability declines.

## 2. Theoretical Foundation

### 2.1. Multimodal Intent Recognition

As the cognitive core of natural language understanding and human–computer interaction (HCI) systems, intent recognition plays a central role in accurately interpreting users’ underlying intentions. Research in this area has undergone a paradigm shift from shallow feature engineering to deep semantic representation. Early approaches were primarily focused on text and used traditional machine learning algorithms such as Naive Bayes, logistic regression, and support vector machines [3]. These methods provide relatively limited inference by manually extracting keywords and syntactic templates. While they can be advantageous for deployment in small-scale, domain-specific applications with limited computational resources, their strong dependence on manual feature design makes it difficult to capture long-range textual dependencies and deeper semantic relationships. With the development of neural networks, deep learning frameworks based on Convolutional Neural Networks (CNNs), Recurrent Neural Networks (RNNs), and large-scale Pre-trained Language Models (PLMs) have substantially expanded the performance limits of text-based intent recognition, equipping models with enhanced nonlinear modeling and adaptive feature-extraction capabilities [8]. However, in real-world open interaction scenarios, human intent expression is often unstructured and intertwined with multimodal cues. Isolating text symbols alone often results in the loss of important emotional and motivational cues hidden in acoustic tone, facial expressions, or body language, making text-only models more susceptible to misinterpretation when encountering ambiguous or emotionally rich inputs. To overcome the perceptual constraints of unimodal methods, multimodal intent recognition has emerged as an advanced framework capable of jointly decoding textual and non-verbal information, and has become a widely used approach for comprehensive inference of user intent.

Throughout the development of multimodal intent recognition, effective alignment and deep integration of heterogeneous data have remained major challenges limiting performance gains. To address temporal misalignment across different modality sequences, Tsai et al. [16] introduced the cross-modal Transformer architecture, which enables adaptive information exchange between modalities by incorporating latent feature mapping. Subsequently, to further reduce redundancy during heterogeneous fusion, attention mechanisms have been extensively incorporated into multimodal network structures. Among these, the text modality, owing to its dense and precise semantics, has gradually emerged as the central guide for cross-modal interaction. For example, Li et al. [8] designed a text-guided cross-attention network that utilizes text semantics as query vectors to selectively filter redundant background noise in non-text modalities; building upon this, the SaliText framework was later developed, which enhances salient semantic regions and improves structured feature representation by integrating modality saliency awareness with textual guidance [7]. Furthermore, Kim et al. [17] explored a dynamic feature fusion mechanism that couples self-attention with cross-attention to balance intermodal representation capacity, providing an approach for extracting robust intent representations.

Despite the continuous development of new fusion techniques, the real-world deployment and generalization of multimodal intent recognition systems remain constrained by the limited availability of realistic datasets. Early multimodal studies were largely conducted in controlled laboratory environments. For example, Trick et al. [18] attempted to combine multiple signal sources such as speech, gestures, and gaze by training independent classifiers and cascading their outputs, while Maharana et al. [19] developed a real-time vision-text multimodal intent detection model tailored for educational applications. These studies demonstrated the feasibility of multi-channel collaboration in reducing interaction uncertainty. However, due to the overly idealized nature of their data acquisition environments, model generalization and performance declined markedly when applied to complex real-world scenarios characterized by background noise and incomplete features. To overcome this limitation, Zhang et al. [20] constructed and open-sourced the MIntRec benchmark dataset. This dataset reflects a shift toward complex, everyday real-world interactions, providing a useful evaluation benchmark for validating multimodal models in unstructured environments. As a result, it has facilitated the transition of multimodal intent recognition from theoretical research toward practical applications.

### 2.2. Multimodal Feature Representation and Fusion Mechanisms

In multimodal language understanding tasks, the ability to obtain high-quality unimodal representations and to design effective cross-modal fusion strategies directly influences attainable model performance.

At the feature representation level, early studies primarily relied on manually extracted acoustic and visual features. With the advancement of pre-training techniques, general-purpose models trained on large-scale datasets (such as BERT for textual data) have gradually become the standard approach for feature extraction [6]. However, given the large number of fine-grained and easily confused categories in intent recognition, general-purpose pre-trained text representations often lack sufficient task-specific discriminative power. In recent years, parameter-efficient prompt tuning methods have provided a promising solution to this problem [13]. By introducing a small set of learnable continuous vectors (soft prompts) at the beginning of the input sequence, the model can elicit task-relevant semantic knowledge without modifying the pretrained parameters. Recent findings suggest that integrating prompt learning into large language models or multimodal recommendation systems can significantly improve the ability of models to capture complex implicit user intentions [21]. This strategy, which leverages semantic prior knowledge, forms a critical theoretical basis for the soft prompt (IP-SPT) constructed in this study through the extraction of intent prototype words.

To reduce the negative impact of unimodal degradation on the overall system, adaptive gated aggregation mechanisms have increasingly attracted attention. The fundamental idea of gating mechanisms is analogous to Mixture-of-Experts (MoE) systems, where a routing network dynamically assigns weights to different input branches [12]. However, during training, traditional gated networks are prone to “routing collapse” caused by the early dominance of a single modality, where the gate persistently suppresses weaker modalities, effectively reducing the multimodal framework to a single-modality system. In interdisciplinary areas such as physiological signal monitoring, where robustness is critical, researchers have proposed the use of the Signal Quality Index (SQI) to explicitly quantify data reliability and guide the dynamic allocation of fusion weights, thereby effectively reducing interference caused by signal fluctuation [14]. Accordingly, hybrid networks capable of explicit quality evaluation and adaptive routing are needed to prevent deadlocks in elderly-care intent recognition scenarios characterized by uncontrollable noise. This requires the model not only to quantify the current quality of text and audio adaptively, but also to adjust modality dependencies automatically in extremely imbalanced environments, thereby achieving a balance between suppressing noise propagation and avoiding routing collapse.

### 2.3. Cross-Attention Mechanisms and Contrastive Learning

In multimodal understanding and robust representation learning, cross-attention mechanisms and contrastive learning have gradually become essential techniques for addressing modal heterogeneity and noise interference.

Attention mechanisms were originally developed to enable models to dynamically focus on the most informative local features within input sequences. With the advancement of multimodal architectures, traditional self-attention has been extended to cross-modal cross-attention. The fundamental principle involves using features from one modality as query vectors to retrieve and aggregate key-value pairs from another modality, thereby achieving cross-modal semantic alignment and information adaptation [16]. However, in practical scenarios, when a specific modality is heavily corrupted by external noise, direct symmetric attention aggregation often fails to fully filter irrelevant features and may even amplify the propagation of noise into deeper network layers [11]. Consequently, some studies have explored asymmetric filtering strategies dominated by high-quality modalities. Inspired by this idea, this paper introduces a unidirectional text-guided cross-attention mechanism [8]. This mechanism uses stable textual features as guidance to perform denoising and refinement of degraded acoustic features, effectively removing redundant background noise at the representation level and providing a high-quality representational foundation for the subsequent gated fusion module.

Complementarily, contrastive learning, as an effective representation learning framework, aims to minimize the distance between similar samples while maximizing the separation between dissimilar samples in feature space by constructing positive and negative pairs. Recently, supervised contrastive learning (SCL) has been proposed and widely applied in downstream classification tasks. Compared with traditional self-supervised contrastive learning, SCL fully exploits label information, treating samples belonging to the same class as positive pairs, thereby more effectively enhancing intra-class compactness and inter-class separability [15]. In multimodal intent recognition tasks, direct fusion of heterogeneous features often introduces distributional shifts in the semantic space and may obscure originally clear core intent cues [22]. By incorporating a contrastive learning framework, the model can encourage cross-modal representations to encode more consistent and essential semantic features [23]. This paper introduces supervised contrastive learning into the final optimization stage of the multimodal fusion architecture. By constructing contrastive relationships between clean text features and fused multimodal features, it not only enhances the discriminative capability of feature boundaries but also provides an additional constraint for the model in severely degraded environments, thereby improving intent-recognition robustness and feature stability.

## 3. Method

### 3.1. Overall Architecture

**Figure 1:**
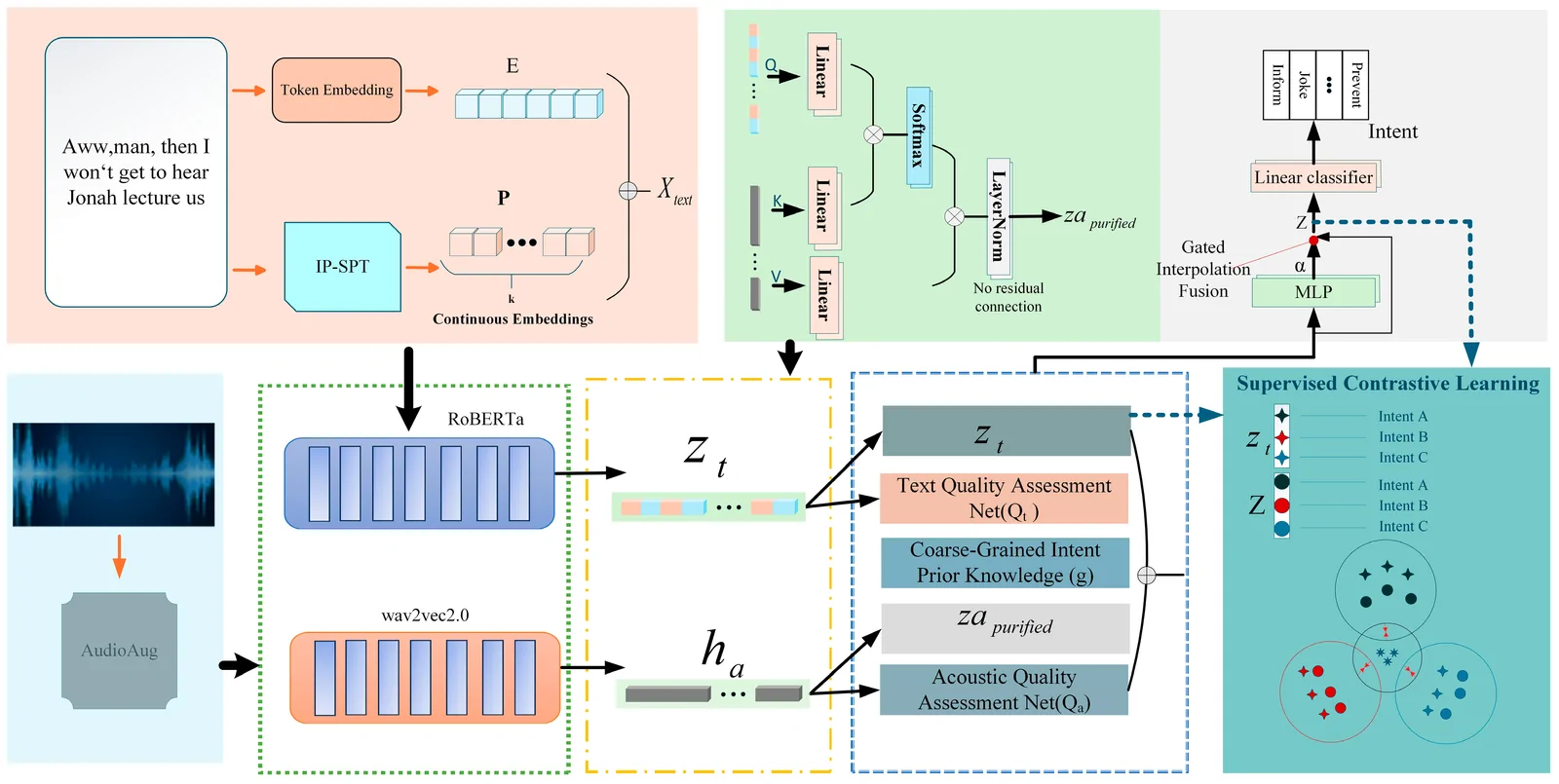
Overall Structure Diagram of the Model.

To address the prevalent problem of multimodal feature degradation in home-based elderly care scenarios—arising from unclear speech among older users and environmental noise interference—this paper presents a robust multimodal intent recognition framework tailored for degraded environments. As shown in Figure 1, the framework first enhances text priors through Intent Prototype Soft Prompting (IP-SPT) to establish semantic anchors; second, it deploys a residual-free cross-modal attention mechanism to use text guidance for unidirectional refinement of degraded audio and to block noise-propagation pathways; subsequently, the RTDF mechanism achieves adaptive routing by evaluating modality quality in real time; when severe acoustic degradation is identified, it triggers modality switching; finally, during the joint optimization stage, supervised contrastive learning (SCL) is incorporated to reduce semantic drift within the representation space.

This framework comprises four principal modules: feature extraction and enhancement, cross-modal interaction and denoising, dynamic fusion, and joint optimization and classification. Table 1 presents a summary of the notations adopted in this study.

1. Feature Extraction and Enhancement

The system accepts paired text sequences and acoustic signals as inputs. On the textual side, to address the limited discriminative ability of pre-trained language models for fine-grained, semantically similar intents, the Intent Prototype Soft Prompt (IP-SPT) mechanism is introduced. A soft prompt vector derived from the global statistical distribution is concatenated with the original textual features and then fed into RoBERTa [24], thereby extracting a clean text representation enriched with task-specific prior knowledge. On the acoustic side, considering the prevalence of environmental noise and speech degradation in home-based elderly care scenarios, an Audio Robustness Enhancement (AudioAug) module is adopted. During training, this module performs random masking and noise injection on the original acoustic sequences across both temporal and frequency domains. Subsequently, Wav2Vec 2.0 [25] is used to extract low-level acoustic representations with enhanced noise-robust generalization capability under such perturbations.

2. Cross-Modal Interaction and Denoising

To address redundancy among heterogeneous modalities and audio distortion under extremely low signal-to-noise ratios, this stage abandons the conventional bidirectional attention mechanism—which is prone to cross-modal noise contamination—and instead adopts a unidirectional text-guided cross-attention network. This network strictly uses clean text features as query vectors (Query) to perform semantic retrieval and local refinement within the corrupted acoustic feature space (Key-Value). Specifically, to fully eliminate the direct noise-propagation paths, this module removes the identity mapping of acoustic features (i.e., adopts a residual-free design), ensuring that all acoustic information entering deeper layers undergoes strict filtering guided by textual semantics.

3. Dynamic Fusion

After obtaining refined unimodal representations, to mitigate the “routing collapse” issue caused by gated networks’ excessive reliance on a single modality under extreme noise, this paper proposes a Quality-Type Dynamic Fusion mechanism (RTDF). Unlike conventional static concatenation, RTDF incorporates lightweight quality probes in the cross-modal feature space to evaluate the reliability of the current text and denoised audio signals in real time. This mechanism couples coarse-grained intent priors with unimodal representation states to adaptively compute fusion weights. When severe audio degradation is detected, the RTDF mechanism switches from weighted fusion by setting the text weight to 1.0, thereby triggering a modality switch. This approach effectively blocks noise interference and shifts decision-making to the text modality alone, ensuring model robustness under adverse acoustic conditions.

4. Joint Optimization and Classification

The final fused representations are passed into a classifier to generate fine-grained intent predictions. To further address the semantic drift introduced during heterogeneous feature fusion, this stage incorporates a supervised contrastive learning (SCL) objective in addition to the primary cross-entropy loss. This objective treats pure textual features as semantic anchors in the representation space, encouraging multimodal fused features to align with the corresponding class centers represented by text features. Combined with a gated regularization term designed to prevent early routing collapse, the overall framework supports end-to-end robust optimization.

### 3.2. Multimodal Feature Extraction and Enhancement

#### 3.2.1. Text Feature Extraction Based on IP-SPT

In daily elderly care interactions, many fine-grained intents are semantically similar and easily confused. To enhance the language model’s ability to differentiate such intents, an Intent Prototype Soft Prompting (IP-SPT) mechanism is introduced at the text input stage. First, for each fine-grained intent category c in the training dataset, the *χ*^2^ statistic between each word ω and that category is computed to measure the discriminative association of the word with that category:

(1) $$\chi^{2}\left(\omega ,c\right)=\frac{N{\left(AD-BC\right)}^{2}}{\left(A+C\right)\left(B+D\right)\left(A+B\right)\left(C+D\right)}$$

Here, *A* denotes the number of samples containing the word ω and belonging to category c; *B* is the number of samples that contain the word *ω* but do not belong to category c; *C* is the number of samples that do not contain the word *ω* but belong to category c; and *D* is the number of samples that do not contain the word ω and do not belong to category c. *N* is the total number of samples.

After ranking the statistical scores, the top k most discriminative prototype words are selected and mapped into a trainable continuous soft prompt vector $P\in {\mathbb{R}}^{k\times d}$ (in this paper, we set k=5 by default, and d is the dimension of the hidden layer). The continuous embeddings P of the constructed k intent prototype soft prompts are concatenated with the embeddings E of the tokenized input text sequence along the sequence dimension, and the combined representation is then fed into the RoBERTa model. To maintain computational efficiency, the core parameters of RoBERTa are frozen, while only the soft prompts and subsequent layers are trained. The feature-extraction process can be expressed as:

(2) $$X_{text}=\left[P;E\right]$$

(3) $$z_{t}=RoBERTa{\left(X_{text}\right)}_{\left[CLS\right]}$$

Here, $X_{text}\in {\mathbb{R}}^{d}$ represents the extracted deep semantic feature vector of the text.

#### 3.2.2. Robust Audio Feature Extraction Based on AudioAug

On the acoustic side, an AudioAug-based robustness enhancement strategy is used to apply stochastic transformations to input audio waveforms during the training phase. These transformations include Gaussian white noise injection, time-stretching for speech rate variation, and random masking across both feature dimensions and temporal steps. The augmented acoustic sequences are then processed using a pre-trained Wav2Vec 2.0 model, followed by linear projection and aggregation into audio feature vectors $h_{a}\in {\mathbb{R}}^{d}$:

(4) $$h_{a}=W_{a}\cdot Wav2Vec2\left(AudioAug\left(X_{audio}\right)\right)+b_{a}$$

Here, $W_{a}$ and $b_{a}$ denote the learnable parameters of the linear projection layer, used to align the feature dimensions with those of the text modality. Similarly, the core parameters of Wav2Vec 2.0 remain fixed during training.

### 3.3. Text-Guided Cross-Modal Cross-Attention Denoising

Because the acoustic feature $h_{a}$ contains residual environmental noise that cannot be fully removed by a basic encoder, a unidirectional text-guided cross-modal attention mechanism is introduced. This mechanism employs the clean text feature $z_{t}$ as the query vector to retrieve relevant key–value representations from the acoustic feature $h_{a}$, thereby enabling deep cross-modal refinement:

(5) $$Q=z_{t}W_{Q},\;K=h_{a}W_{K},\;V=h_{a}W_{V}$$

(6) $$za_{purified}=LayerNorm\left(Soft\max\left(\frac{QK^{T}}{\sqrt{d_{k}}}\right)V\right)$$

Here, $W_{Q}$, $W_{K}$, and $W_{V}$ denote the learnable projection matrices of the attention mechanism, and $d_{k}$ represents the scaling factor. To prevent noise propagation at the feature level, residual connections for acoustic features are intentionally removed in this layer, ensuring that the output $za_{purified}$ represents a denoised acoustic feature filtered through textual guidance.

### 3.4. Quality-Type Dynamic Fusion (RTDF)

After the purified unimodal features are obtained, a quality-type dynamic fusion gating mechanism is introduced to avoid “routing collapse” under severe noise conditions. This mechanism utilizes a lightweight quality-aware network to estimate reliability scores $q_{t}$ and $q_{a}$ for the text and audio modalities of the current sample:

(7) $$q_{a}=\sigma \left(W_{qt}z_{t}+b_{qa}\right)$$

Here, σ denotes the sigmoid activation function. Subsequently, the gating network concatenates the textual features, denoised acoustic features, quality scores, and the one-hot encoded coarse-grained intent category g, and applies a multi-layer perceptron (MLP) to dynamically compute the text-modality weighting coefficient α:

(8) $$\alpha =\sigma \left(MLP\left(\left[z_{t},za_{purified},q_{t},q_{a},g\right]\right)\right)$$

Using the computed weighting factor α, a convex combination of the two modalities is performed to obtain the final fused feature representation z:

(9) $$z=\alpha \cdot z_{t}+\left(1-\alpha \right)\cdot za_{purified}$$

### 3.5. Joint Optimization and Classification

The fused features z are first fed into a linear classifier to estimate the probability distribution $\hat{y}$ of the user’s fine-grained intent:

(10) $$\hat{y}=Soft\max\left(W_{c}z+b_{c}\right)$$

Here, $W_{c}$ and $b_{c}$ denote the trainable parameters of the classification layer.

During the training stage, to ensure the stability of feature representations and the separation of intent boundaries in extremely noisy environments, this paper proposes a three-part optimization objective:

1. Classification Main Objective Loss

The standard cross-entropy loss is adopted as the primary objective for intent classification:

(11) $$L_{ce}=-\sum_{i=1}^{C}y_{i}\log\left({\hat{y}}_{i}\right)$$

2. Gated Regularized Loss

To prevent the RTDF gating network from becoming prematurely biased toward a single modality due to accidental dominance during early training, a small gated regularization term is incorporated:

(12) $$L_{gate}=\frac{1}{F}\sum_{i=1}^{F}{\left(\alpha_{i}-0.5\right)}^{2}$$

where F denotes the batch size. This regularization term introduces a weak gradient signal, ensuring that the attention-based denoising module remains sufficiently activated during the initial training phase.

3. Supervised Contrastive Learning Loss

Direct fusion of heterogeneous modalities may introduce distributional inconsistencies. To mitigate this issue, a supervised contrastive learning mechanism is incorporated. This mechanism treats clean text features $z_{t}$ as anchors and fused features z as contrastive representations, ensuring that samples belonging to the same intent category remain close in the representation space:

(13) $$L_{scl}=\sum_{i=1}^{B}\frac{-1}{\left|P\left(i\right)\right|}\sum_{p\in P\left(i\right)}\log\frac{\exp\left(\upsilon_{i}\cdot \upsilon_{p}/\tau \right)}{\sum_{a\in A\left(i\right)}\exp\left(\upsilon_{i}\cdot \upsilon_{a}/\tau \right)}$$

Here, *v* denotes the set of projection features used for comparison; $A\left(i\right)$ represents the indices of all samples in the batch except the anchor i; $P\left(i\right)$ denotes the set of positive samples sharing the same intent label as anchor i; and τ denotes the temperature coefficient.

4. Total Loss Function

Finally, the entire multimodal intent recognition framework is trained end-to-end by jointly optimizing the three losses described above:

(14) $$L_{total}=L_{ce}+\lambda_{gate}L_{gate}+\lambda_{scl}L_{scl}$$

In particular, $\lambda_{gate}$ and $\lambda_{scl}$ represent hyperparameters for balancing different loss components. Under this objective, the model achieves accurate classification while maintaining robustness under severely degraded conditions.

AI Disclosure: While writing this article, the authors used ChatGPT (GPT-5, OpenAI, 2025) to assist with the generation of some of the text; this generative AI tool was not used to generate data, process data, or create charts.

## 4. Experimental Section

### 4.1. Dataset

MIntRec [20] is a multimodal intent recognition dataset derived from the TV series “Supermarket”, consisting of 2224 samples across three modalities: text, audio, and video. At the coarse-grained level, the dataset is divided into two primary categories: “Expressing Emotions or Attitudes” and “Achieving Goals”; at the fine-grained level, it is further partitioned into 20 specific intent classes (e.g., complaining, requesting help, seeking advice, refusing, comforting, etc.), covering representative intent types in everyday human–computer interaction contexts relevant to elderly care. Detailed statistical information for this dataset is presented in Table 2.

Regarding data partitioning, this study strictly followed the official MIntRec protocol, dividing the dataset into a training set (1334 samples), a validation set (445 samples), and a test set (445 samples) in a 3:1:1 ratio, as shown in Table 3. Given the limited availability of multimodal intent datasets specifically designed for the elderly population, MIntRec was selected as the evaluation benchmark due to its fine-grained intent annotations and standardized data partitioning. It should be noted that MIntRec was not collected exclusively from elderly users. Therefore, controlled acoustic perturbations were introduced to create standardized acoustic degradation conditions reflecting challenges that may occur in older-adult speech and to evaluate the model’s robustness when acoustic reliability is reduced.

### 4.2. Data Preprocessing and Harsh Environment Simulation

1. Text and Audio Preprocessing

For the text modality, the official pre-trained RoBERTa tokenizer is employed to process the raw transcription text, after which sequences are truncated or padded to a fixed length to obtain the input tokens and attention masks required by the model; for the audio modality, to eliminate scale inconsistencies in audio features across samples, accelerate training convergence, and avoid gradient instability, standard Z-score normalization is used to process the extracted acoustic features. Specifically, the mean *μ* and standard deviation *σ* of the audio features are first computed across all dimensions from the training set. These global statistics are then applied consistently to normalize audio features in the training, validation, and test sets:

(15) $$x_{norm}=\frac{x-\mu }{\sigma +\varepsilon }$$

Here, $\varepsilon =10^{-6}$ is a smoothing constant introduced to prevent division by zero.

2. Simulation of Harsh Environments for Elderly Care Settings

The original MIntRec dataset was collected under relatively clean and controlled recording conditions and was not specifically designed for elderly users. Therefore, the perturbation strategy adopted in this study is not intended to reproduce physiologically authentic speech of older adults, but to create controlled, reproducible degradation conditions to evaluate the model’s robustness when acoustic reliability is reduced. Previous studies have highlighted changes in speech timing associated with aging, including slower speech rate and increased pauses; however, real-world interactions in elderly care may also be affected by background noise and incomplete acoustic information. Based on these considerations, this paper explores three representative types of acoustic degradation: temporal changes, background noise interference, and partial loss of acoustic information.

Speech Rate and Temporal Variability: Following the speech rate perturbation strategy commonly used in speech recognition [26], we apply modest time scaling using a factor randomly sampled from the range [0.95, 1.05] to introduce temporal variability while avoiding excessive distortion of semantic information.

Background Noise Disturbance: Gaussian noise with a signal-to-noise ratio (SNR) range of 15–30 dB was introduced to evaluate the model’s robustness when acoustic reliability is reduced. This setup is intended to provide a controlled degradation condition relevant to an elderly-care environment.

Partial Acoustic Information Masking: Following the basic approach of SpecAugment [27], we randomly mask 5–15% of the time steps and 5–10% of the feature channels to simulate conditions where part of the acoustic information is unavailable or unreliable.

Through these perturbations, a standardized, reproducible degraded-input condition was established, and the original MIntRec dataset was transformed into a more challenging “Degraded Multimodal Intent Dataset”. The purpose of this setup is to evaluate the model’s robustness under conditions representing reduced acoustic fidelity relevant to older-adult interaction, rather than to fully replicate real-world speech of older adults. Real-world elderly speech may also involve significant inter-speaker variability, variations in articulation and voice quality, and more complex environmental factors, all of which are beyond the scope of this controlled simulation.

### 4.3. Baseline

To ensure consistency and fairness in the comparison, this paper selected baseline methods spanning unimodal recognition, traditional multimodal fusion, and Transformer-based cross-modal interaction to evaluate the performance of different fusion strategies under a unified experimental setup. In recent years, several new dynamic fusion and cross-modal interaction methods have been proposed in the field of multimodal intent recognition; however, some of these methods differ from this study in terms of input-modality combinations, feature-representation methods, and experimental evaluation protocols, making direct comparison with their published results difficult under the controlled input degradation conditions established in this study. Therefore, this study uses representative baselines that can be reproduced and compared fairly under a unified data partition, input modalities, and evaluation conditions.

To comprehensively evaluate the effectiveness and robustness of the model proposed in this paper in degraded multimodal environments, six representative baseline models were selected, covering unimodal approaches, traditional multimodal fusion methods, and advanced attention-based multimodal architectures for comparison:

Text-Only: Utilizes only textual modality data, extracts [CLS] representations using a pre-trained RoBERTa model, and feeds them into a multi-layer perceptron (MLP) for intent classification.

Audio-Only: Relies exclusively on audio modality data; acoustic features are extracted using Wav2Vec 2.0, followed by pooling, and then passed into a classifier for prediction.

Concat-MLP [28]: As a fundamental early fusion strategy in multimodal learning, this method independently extracts text and audio features, concatenates them at the feature level, and inputs the combined vector into an MLP classifier.

Late-Fusion [29]: A classical decision-level fusion approach, where text and audio modalities are processed through separate classifiers to produce probability distributions, and the final prediction is obtained by computing a weighted average of these outputs.

MulT [13]: A representative cross-modal Transformer-based architecture, which employs paired directional cross-attention to implicitly align and adapt features across different modality sequences.

MAG-BERT [30]: An effective framework proposed for embedding multimodal information within pre-trained language models. Through a multimodal adaptation gate, this model dynamically injects non-linguistic information as additional features into text representations at different layers of the Transformer architecture.

### 4.4. Test Environment and Evaluation Metrics

To further evaluate the model complexity and computational requirements, this paper provides statistics on parameter count, inference time, and resource requirements. The total number of parameters in the complete model is approximately 128.54 M, of which the modules added in this paper account for about 3.90 M. This corresponds to an increase of approximately 3.13% in parameter count compared to the baseline RoBERTa-base, indicating that the additional parameter overhead introduced by the new modules is limited. When tested on an NVIDIA RTX 4060 GPU (Gigabyte, Shenzhen, China) with a batch size of 1, the model’s average inference time per sample was 8.90 ms. The experimental environment consisted of an Intel Core i5-13400F CPU (Intel, Shenzhen, China), an NVIDIA RTX 4060 GPU, CUDA 12.1, and PyTorch 2.1.0. The reported inference times represent the forward-pass computation after text and pre-extracted acoustic features are provided to the model; they do not include the time required for upstream acoustic feature extraction.

To reduce the influence of random initialization and stochastic optimization on the experimental results, this paper conducted independent replicate experiments using five different random seeds (42, 43, 44, 45, and 46) for the proposed method and all baseline methods, and computed the mean and standard deviation of Accuracy, Macro-F1, and Weighted-F1 across five runs. Furthermore, a two-sided paired t-test was performed on the paired experimental results obtained under the same random seeds to evaluate the statistical reliability of the observed differences in performance. The Holm method was used for multiple-comparison correction, and the corrected value of $p_{Holm}<0.05$ was adopted as the criterion for statistical significance.

To maintain consistency with prior studies, a consistent set of evaluation metrics is used to comprehensively assess model performance, including accuracy (Acc), precision (P), recall (R), macro-F1 score, weighted precision (WP), and weighted F1 score (WF1). Given the numbers of true positives (*TP*), false positives (*FP*), true negatives (*TN*), and false negatives (*FN*), the corresponding evaluation metrics are defined as follows:

(16) $$Accuracy=\frac{TP+TN}{TP+TN+FP+FN}$$

Precision and Recall: Precision measures the proportion of samples that the model predicts to belong to a certain intent category that actually belong to that category; recall measures the proportion of all samples that actually belong to a certain intent category that are correctly identified by the model.

(17) $$Precision=\frac{TP}{TP+FP}$$

(18) $$Recall=\frac{TP}{TP+FN}$$

Class-wise F1-Score: For a given intent category i, its F1 score is the harmonic mean of the precision and recall for that category.

(19) $$F1_{i}=\frac{2\times Precision\times Recall_{i}}{Precision_{i}+Recall_{i}}$$

Macro-F1 Score: The arithmetic mean of F1 scores across all categories, treating each category equally and reflecting the model’s ability to recognize minority classes (rare intents).

(20) $$Macro\text{-}F1=\frac{1}{C}\sum_{i=1}^{C}F1_{i}$$

Weighted Metrics: The scores are averaged across categories using the proportion of actual samples in each category as the weight, thereby reducing evaluation bias caused by the long-tail distribution of the dataset:

(21) $$Weighted\text{-}Precision\left(WP\right)=\sum_{i=1}^{C}\omega_{i}\cdot Precision_{i}$$

(22) $$Weighted\text{-}F1\left(WF1\right)=\sum_{i=1}^{C}\omega_{i}\cdot F1_{i}$$

Here, c represents the total number of intent categories (c = 20 for this task), and $\omega_{i}$ represents the actual proportion of samples in the test set belonging to the ith intent category.

## 5. Results and Analysis

To systematically evaluate the overall performance of the proposed multimodal intent recognition framework, which integrates intent prototype soft prompts with dynamic fusion gating (IP-SPT + RTDF), this section presents comparative experiments under both clean and degraded conditions on the MIntRec dataset, conducts ablation studies on key components, and further investigates fine-grained intent recognition through detailed visual analysis.

### 5.1. Analysis of Main Experimental Results: Comparison of Clean and Degraded Environments

**Figure 2:**
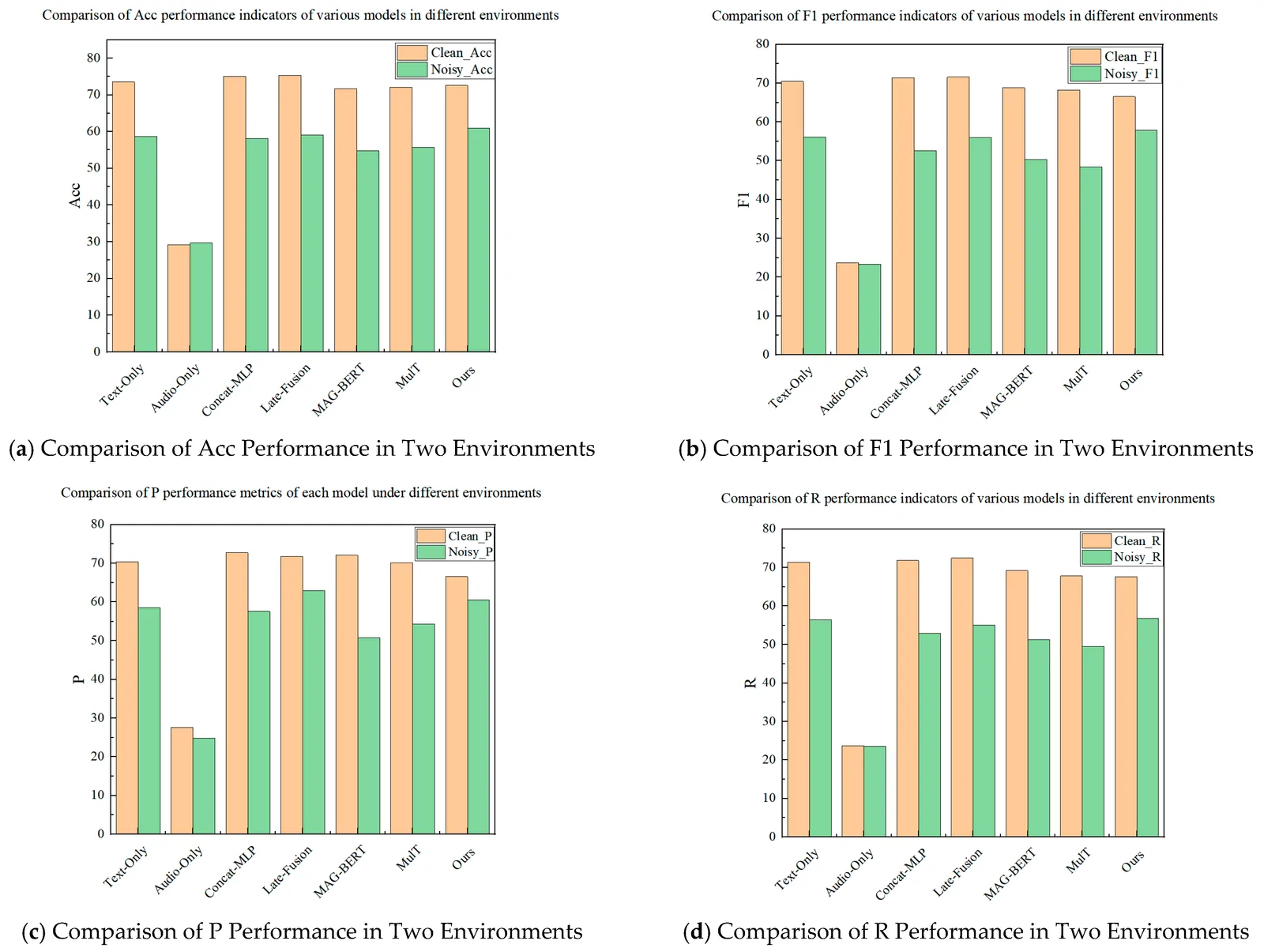

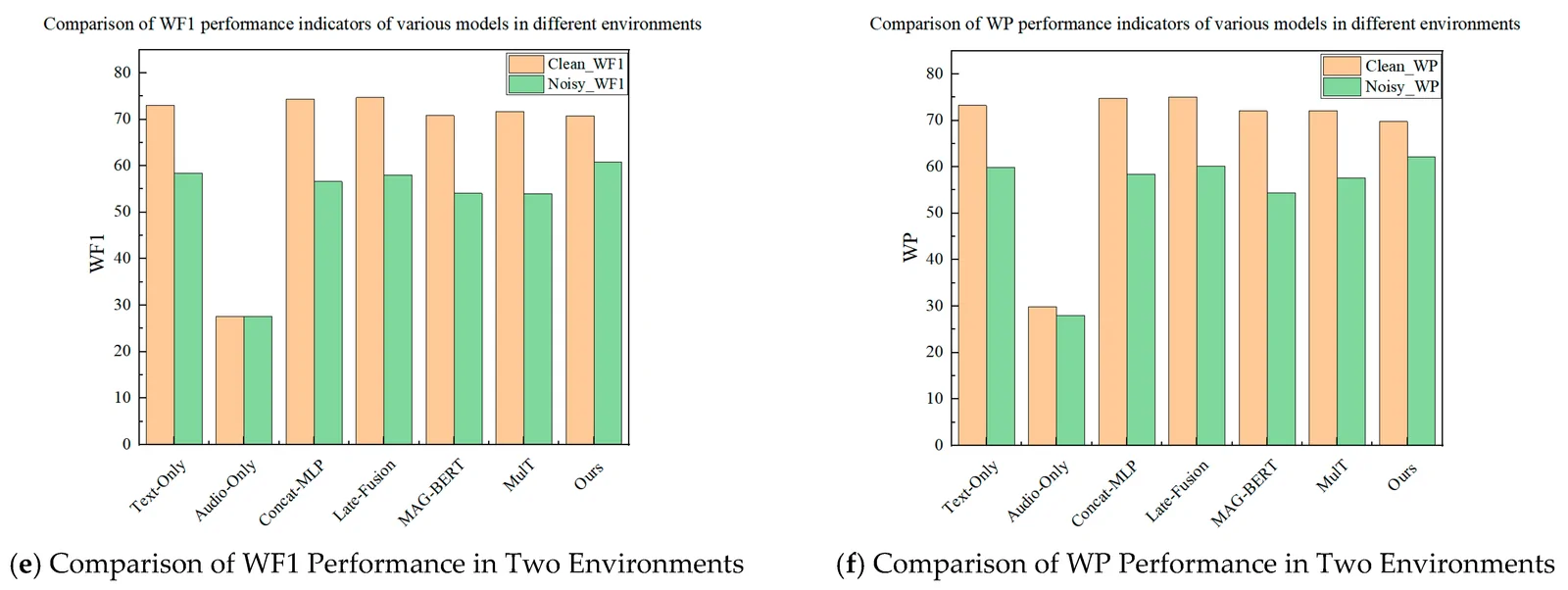
Comparison Chart of Performance Metrics for Various Models Across Different Environments.

Table 4 and Table 5 provide detailed results of key evaluation metrics for each baseline model and the proposed method under two different acoustic environments; the corresponding visual comparisons are presented in Figure 2. Based on these experimental results, the following two principal conclusions are drawn.

Benchmark Performance in a Clean Environment: Under a clean test setting without environmental disturbances, traditional multimodal fusion methods exhibit strong overall performance. Specifically, the weighted F1 score (WF1) of Late-Fusion and Concat-MLP reached 74.70% and 74.35%, respectively, with accuracy (Acc) values of 75.28% and 75.06%. These results indicate that, under ideal conditions with relatively clean acoustic features, simple feature-level concatenation or decision-level weighting strategies are sufficient to support intent classification effectively. In contrast, our model achieved a WF1 of 70.75% and an accuracy of 72.58% in a clean environment, while maintaining comparable performance. Its metrics are slightly lower than those of some traditional fusion methods, primarily because of the “regularization trade-off” phenomenon during model training. Since this study incorporates substantial degraded-data augmentation (AudioAug) and dynamic gating regularization constraints, these mechanisms partially limit the model’s tendency to overfit the clean acoustic distribution. This design entails a modest reduction in fitting accuracy under ideal conditions in exchange for broader generalization when the model encounters real-world, complex interactive environments.

Performance Degradation and Robustness Validation in Degraded Environments: When the testing conditions were switched to a degraded environment simulating real-world home care scenarios (introducing disturbances such as background noise and speech rate variation), the models showed clearly different degradation patterns, and baseline performance declined when acoustic features were distorted. For example, the WF1 score of Concat-MLP decreased substantially from 74.35% to 56.63%; similarly, the MulT model, which is based on a cross-modal Transformer, was strongly affected. Due to its unconstrained attention mechanism that allows noise to propagate across modalities and disrupt global semantics, its WF1 score fell from 71.63% to 53.98%. Noise robustness of the proposed model: Unlike the significant performance degradation observed in baseline models, the proposed model maintained comparatively stable performance under degraded conditions. Its accuracy was 60.90%, with a weighted F1 score of 60.80% and a macro-F1 score of 57.90%. More importantly, the overall performance reduction remained within 10%. This relatively stable performance indicates that the text-guided cross-attention mechanism effectively filters acoustic disturbances, while the RTDF gating module adaptively adjusts fusion weights when modal quality deteriorates. Together, these two components support the reliability of intent recognition under complex environmental noise.

To further evaluate the stability and statistical reliability of the experimental results, this study conducted independent replicate experiments using five different random seeds. The mean Accuracy, Macro-F1, and Weighted-F1 of the proposed method were 59.55 ± 1.46%, 55.88 ± 0.58%, and 58.66 ± 0.65%, respectively, indicating that the model maintains relatively stable performance across different random initializations. Compared with the best-performing competing baselines for each evaluation metric, the proposed method achieved higher mean performance for all three metrics. Furthermore, a two-sided paired t-test was conducted using paired results obtained with the same random seeds, and the Holm method was used for multiple-comparison correction. The results indicated that the performance improvements of our method in Accuracy, Macro-F1, and Weighted-F1 were statistically significant ($p_{Holm}<0.05$), after Holm correction, supporting the statistical reliability of the observed performance gains across different random initializations.

### 5.2. Analysis of Ablation Experiments

**Figure 3:**
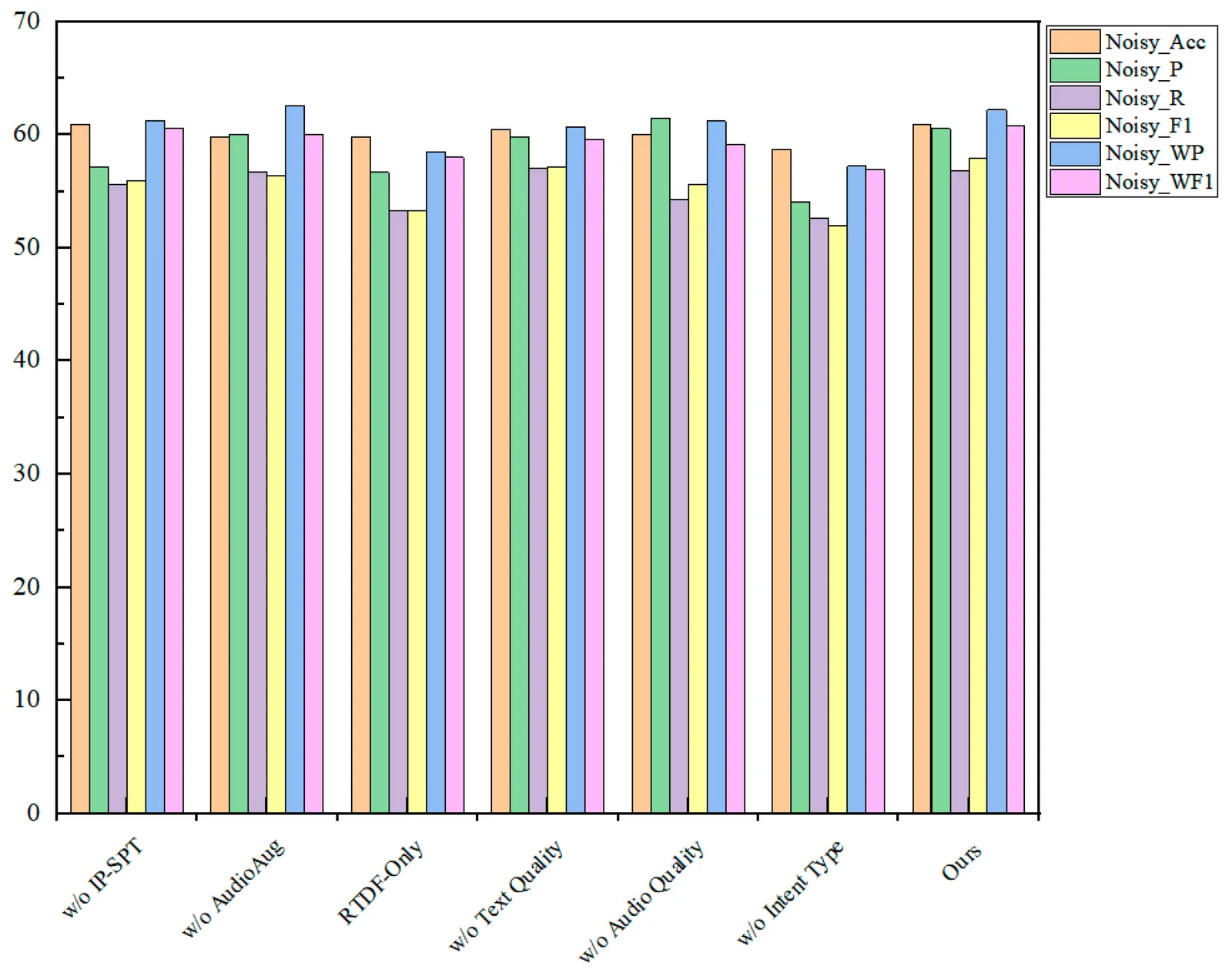
Comparison of Different Performance Metrics in Ablation Experiments.

To systematically assess the effectiveness and individual contributions of each key component in the proposed model, comprehensive ablation experiments were conducted under degraded conditions. By progressively removing or modifying core modules, the performance of different model variants is reported in Table 6, with corresponding visual comparisons shown in Figure 3.

Validation of the Effectiveness of Intent-Based Prompts and Data Augmentation Mechanisms: First, the contributions of the Intent Prototype Soft Prompt (IP-SPT) and Audio Augmentation (AudioAug) modules are examined. When the IP-SPT module is removed (i.e., w/o IP-SPT), the overall accuracy remains relatively stable, but the macro-F1 score decreases from 57.90% to 55.90%. This variation suggests that incorporating high-quality textual prompts before multimodal fusion assists the model in defining clearer semantic boundaries for complex or long-tail intents, thereby improving recognition balance across minority categories. Furthermore, when the audio augmentation module is removed (i.e., w/o AudioAug), the weighted F1 score (WF1) decreases to 59.96%. This reduction occurs because the model is no longer exposed to degraded acoustic distributions during training, resulting in limited generalization capability when encountering noisy conditions in the test set, and consequently leading to reduced robustness against acoustic interference.

Fine-Grained Analysis of the RTDF Gating Mechanism: To verify the effectiveness of the internal design factors of the Quality-Type Dynamic Fusion Gating (RTDF) mechanism, this paper does not reduce it to conventional feature concatenation (as already compared in Section 5.1) but instead performs a detailed ablation analysis on its critical input components. First, after removing the guidance of coarse-grained intent category features (i.e., w/o Intent Type) from the gating module, the model’s WF1 metric decreased significantly to 56.96%. This result supports the earlier design assumption that different broad intent categories (such as emotional expression and task-oriented requests) inherently exhibit different dependencies on text and audio modalities. Incorporating intent prior information enables the gating network to make more accurate routing decisions. Second, when the text-quality (w/o Text Quality) and audio-quality (w/o Audio Quality) inputs were independently removed from the gating network, the WF1 values decreased to 59.59% and 59.15%, respectively. This result indicates that the effectiveness of the RTDF mechanism does not rely solely on the additional linear network layers, but is instead built upon the capture and adaptive adjustment of real-time modal quality features. Without specific quality assessment inputs, the dynamic gating process degenerates into a static weighting scheme, making it difficult to respond effectively when modal degradation occurs.

Verification of Forced Fusion and Modal Contamination: To investigate the cause of gating-induced modality collapse in the main experiment, this paper conducted a supplementary “forced fusion” experiment: by capping the upper bound of α at 0.982 via Logit clipping, the model was forced to retain only 1.8% of the audio features. As reported in Table 7, even this small amount of noise caused the model’s accuracy and F1 score to decline to 54.38% and 45.33%, respectively. These observations indicate that the high-precision semantic space constructed by IP-SPT is highly sensitive to low-quality noise interference. This further supports the role of the RTDF mechanism, which effectively protects the global representation through a modality-switching strategy by proactively identifying contamination risks and suppressing the unreliable audio channel.

### 5.3. Fine-Grained Visual Analysis of Intent and Features

**Figure 4:**
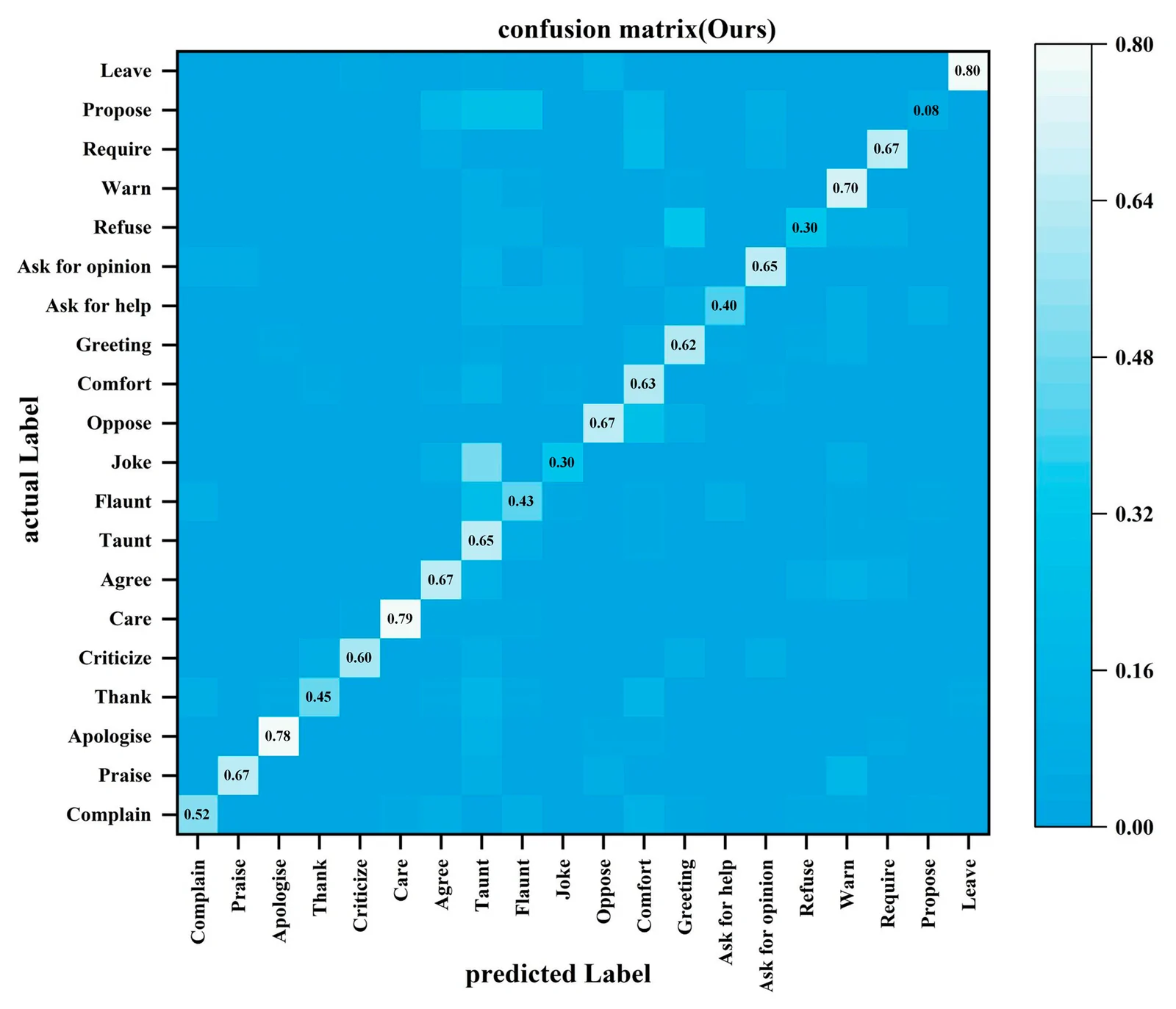
Visualization of the confusion matrix for test accuracy.

**Figure 5:**
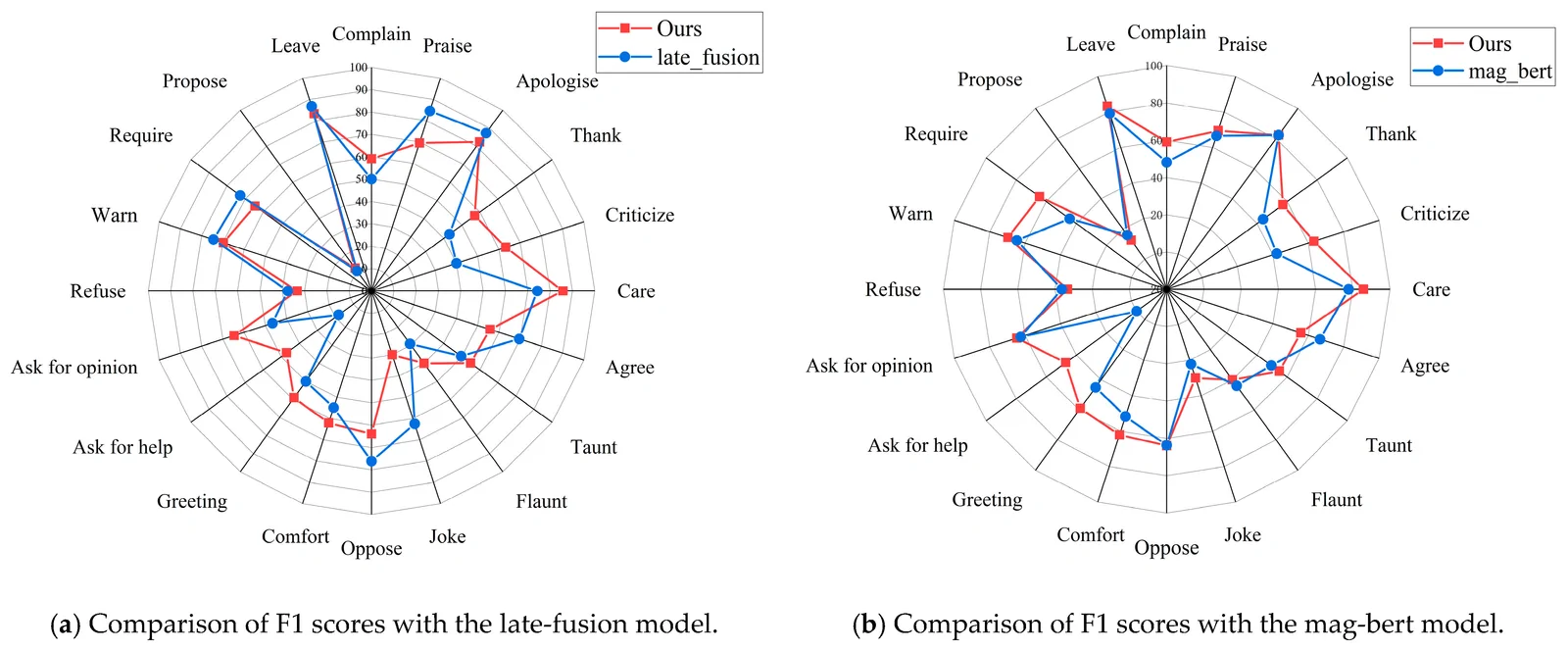

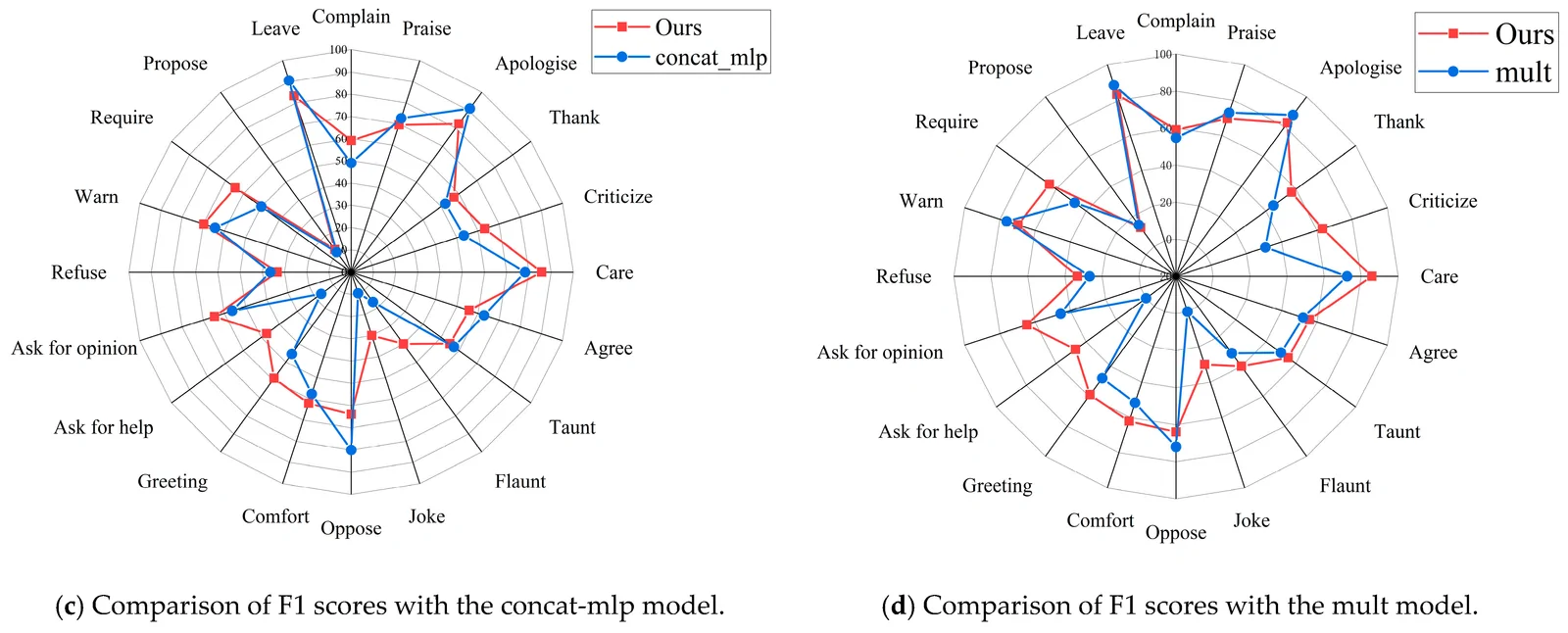
Comparison of Fine-Grained Intent F1 Scores Across Different Models.

Given that this study targets age-friendly interaction scenarios, the recognition performance of specific key intents (such as seeking help or expressing dissatisfaction) is of greater practical relevance than aggregate metrics. Based on the confusion matrix of the complete model (as shown in Figure 4) and the fine-grained F1 scores for each category (as presented in Table 8 and Table 9), a comparative analysis of intent-level F1 performance across different models is illustrated in Figure 5. This study further examines in greater detail the model’s behavior on individual intent categories and potential misclassification patterns.

Performance in Identifying Key Intent Categories: When environmental noise is introduced, baseline frameworks often struggle to capture subtle but essential acoustic cues. A detailed comparison shows that the baseline MulT model exhibits significant limitations in recognizing the critical elderly care intent “Ask for help” (with the F1 score decreasing to 0.00%). In contrast, guided by high-quality prompts generated by the IP-SPT module, the proposed model improved the F1 score for this category to 47.06%, thereby improving the model’s ability to capture emergency assistance intents. Furthermore, in categories such as “Criticize” and “Ask for opinion”—which carry strong emotional connotations and are easily masked by background noise—the F1 scores of the proposed model exceed those of the MulT model by 32.39% and 19.26%, respectively. These results further demonstrate that the proposed framework better preserves fine-grained semantic information and subtle emotional characteristics under complex acoustic conditions.

Analysis of Ambiguous Intent and Model Limitations: Overall, the model exhibits relatively stable performance in recognizing intents with clear feature boundaries. For instance, according to the confusion matrix, the accuracy for “Leave” reaches 80.0%, “Care” achieves 78.9%, and “Apologize” attains 77.8%. This strong inter-class separability is mainly attributed to the Supervised Contrastive Learning (SCL) mechanism incorporated during training. By imposing clustering constraints on samples of the same class in the feature representation space and increasing the distance between samples of different classes, this mechanism effectively widens the decision boundaries between different emotional intents, thereby supporting efficient recognition of most common interaction categories. However, the model still encounters difficulties when dealing with categories that exhibit high semantic similarity. According to the data distribution in the confusion matrix, the “Propose” category exhibits a high rate of misclassification, with 25.0% of samples each incorrectly predicted as “Taunt” and “Flaunt”; the “Joke” category is confused with “Taunt” 50.0% of the time. This error pattern is plausible: when simulated background noise in home environments obscures intonation markers and subtle pitch variations, the mild or humorous acoustic cues required to distinguish “Propose” or “Joke” become weakened. In the absence of clear paralinguistic signals, these intents are more likely to be confused with “Taunt,” which also conveys a non-literal tone. This phenomenon not only reflects the effect of acoustic degradation on fine-grained emotion classification but also suggests a direction for future research, including the integration of visual modalities such as facial micro-expressions or body language as complementary information in scenarios with extremely low signal-to-noise ratios.

## 6. Conclusions

This paper proposes a prompt-guided and quality-aware text–audio intent recognition framework for elderly-oriented interaction under acoustic degradation. The framework integrates Intent Prototype Soft Prompting and Quality-Type Dynamic Fusion to improve recognition robustness when acoustic information becomes unreliable. Specifically, the *χ*^2^-based intent prototype soft prompt module introduces intent-related prior information into the text encoder, thereby enhancing the discriminative capability of textual representations for fine-grained and long-tail intent categories. In addition, the quality-aware dynamic fusion gate estimates the reliability of text and audio features and incorporates intent-type priors to adaptively adjust text–audio fusion weights. When the acoustic modality is severely degraded, the framework can reduce the contribution of unreliable audio features and rely more strongly on textual semantic information.

Experiments on the MIntRec dataset with simulated acoustic degradation demonstrate that the proposed framework achieves improved robustness compared with representative baseline models. The results indicate that quality-aware fusion can alleviate the negative influence of degraded acoustic features and maintain relatively stable performance under challenging acoustic conditions. Fine-grained analysis further shows that the proposed method improves the recognition of assistance-related and semantically subtle intents, such as “Ask for help” and “Ask for opinion,” which are important for elderly-oriented assistive interaction.

Although the proposed framework improves robustness under the simulated acoustic degradation, several issues require further investigation. First, future work will incorporate multiple visual information sources, such as facial expressions, gaze direction, and body posture, to construct a text–speech–vision intent recognition framework. These visual cues may provide complementary information when acoustic cues are weakened by background noise or speech degradation, especially for ambiguous intents such as joking, sarcasm, and emotional expression. Therefore, the current experimental results primarily demonstrate the system’s robustness under controlled acoustic degradation conditions; however, generalization to real-world speech from older populations still requires validation. Additionally, future work will systematically evaluate the model at multiple fixed signal-to-noise ratio (SNR) levels to further investigate the relationship between noise intensity and model robustness.

Second, future research will focus on deployment and validation in real elderly-care environments. The current evaluation is conducted on a benchmark dataset with simulated acoustic perturbations. Further work will collect interaction data from elderly users in realistic home environments and evaluate the model on a physical mobile robot platform. Through real-world deployment, the generalization capability, response latency, and practical reliability of the proposed framework can be further assessed and improved.

## Figures and Tables

**Table 1:** Summary of Basic Symbols.

Symbol	Description
c	Fine-grained intent categories
ω	Per Word
A	The number of samples containing the word ω and belonging to category c
B	The number of samples that contain the word ω but do not belong to category c
C	The number of samples that do not contain the word ω but belong to category c
D	The number of samples that do not contain the word ω and do not belong to category c
N	Total sample size
P	Continuous soft-hinting vectors
E	Embedding vectors
k	Soft prompt
$X_{text}$	Extracted deep, clean semantic feature vectors of the text
$z_{t}$	Output from the RoBERTa model
$W_{a}$, $b_{a}$	Trainable parameters of the linear projection layer
$h_{a}$	Pooled audio feature vectors
$d_{k}$	Scale factor
$W_{Q}$, $W_{K}$, $W_{V}$	Trainable weight matrix for the attention mechanism
$za_{purified}$	A denoised representation fully filtered by text semantics
$q_{t}$, $q_{a}$	Reliability scores for text and audio modalities
σ	Sigmoid activation function/standard deviation
g	Coarse-grained intent categories
$W_{c}$, $b_{c}$	Trainable parameters of the classifier
$L_{ce}$, $L_{gate}$, $L_{scl}$	Classification main objective loss, gated regularization loss, supervised contrastive learning loss
F	Batch size
v	Set of projection features included in the comparison
$A\left(i\right)$	The indices of all samples in the batch except for anchor i
$P\left(i\right)$	The set of positive examples that share the same intent label as anchor i
τ	Temperature coefficient
μ	Smoothing terms that prevent the denominator from becoming zero
TP, FP, TN, FN	True positive, false positive, true negative, false negative
$\omega_{i}$	The true proportion of samples in the test set that belong to Class i

**Table 2:** Statistical Analysis of the MIntRec Dataset.

Item	Statistic
Number of coarse-grained intents	2
Number of fine-grained intents	20
Number of words in the text	15,658
Number of unique words in the text	2562
Average length of text statements	7.04

**Table 3:** Statistical Analysis of Sample Distributions in MIntRec.

Item	Total	Express Emotions or Attitudes
Train	1334	749
Valid	445	249
Test	445	248

**Table 4:** Performance of Each Model Under Standard Conditions.

Method	Acc	F1	P	R	WF1	WP
Text-Only	73.48	70.42	70.34	71.36	73.02	73.26
Audio-Only	29.21	23.72	27.64	23.74	27.61	29.89
Concat-MLP	75.06	71.35	**72.71**	**71.84**	74.35	74.82
Late-Fusion	**75.28**	**71.56**	71.79	72.43	**74.70**	**75.03**
MAG-BERT	71.69	68.79	72.08	69.22	70.81	72.04
MulT	72.13	68.24	70.11	67.78	71.63	72.03
Ours	72.58	66.50	66.53	67.22	70.75	69.76

Bold text indicates the highest score for each model’s corresponding metric.

**Table 5:** Performance of Various Models in Degraded Environments.

Method	Acc	F1	P	R	WF1	WP
Text-Only	58.65	56.13	58.55	54.47	58.37	59.92
Audio-Only	29.66	23.28	24.80	23.52	27.54	27.93
Concat-MLP	58.20	52.59	57.60	52.93	56.63	58.42
Late-Fusion	59.10	55.93	**62.86**	55.16	57.97	60.20
MAG-BERT	54.83	50.35	50.80	51.35	54.06	54.44
MulT	55.73	48.35	54.30	49.50	53.98	57.64
Ours	**60.90**	**57.90**	60.56	**56.86**	**60.80**	**62.17**

Bold text indicates the highest score for each model’s corresponding metric.

**Table 6:** Performance of Core Module Ablation Experiments.

Method	w/o IP-SPT	w/o AudioAug	RTDF-Only	w/o Text Quality	w/o Audio Quality	w/o Intent Type	Ours
Acc	60.90	59.78	59.78	60.45	60	58.65	**60.90**
F1	55.90	56.42	53.26	57.20	55.61	52.00	**58.10**
P	57.20	60.02	56.66	59.84	**61.49**	54.09	60.56
R	55.57	56.68	53.25	**57.09**	54.31	52.65	56.86
WF1	60.62	59.96	57.99	59.59	59.15	56.95	**60.80**
WP	61.24	**62.60**	58.45	60.69	61.18	57.22	62.17

Bold text indicates the highest score for each model’s corresponding metric.

**Table 7:** Performance Evaluation of Forced Fusion and Modality Fusion.

Method	Acc	F1	P	R	WF1	WP
Ours	54.38	45.33	49.43	47.61	50.31	51.84

**Table 8:** Comparison of Fine-Grained Intent F1 Scores Across Different Models.

Intent	Complain	Praise	Apologize	Thank	Criticize	Care	Agree	Taunt	Flaunt	Joke
Concat-MLP	49.06	72.73	90.91	52.38	53.33	78.26	62.86	**57.14**	16.67	10.00
Late-Fusion	50.00	**84.62**	**87.27**	43.14	40.00	74.29	**69.57**	49.61	29.27	**62.50**
MAG-BERT	48.15	66.67	82.35	44.00	42.11	77.78	66.67	49.54	43.90	22.22
MulT	54.84	72.73	87.50	45.00	30.77	72.34	52.17	50.00	31.25	0.00
Ours	**59.09**	69.57	82.35	**57.14**	**63.16**	**85.71**	55.81	54.81	**40.00**	30.00

Bold text indicates the highest score for each model’s corresponding metric.

**Table 9:** Comparison of Fine-Grained Intent F1 Scores Across Different Models.

Intent	Oppose	Comfort	Greeting	Ask for Help	Ask for Opinion	Refuse	Warn	Require	Propose	Leave
Concat-MLP	**80.00**	57.58	45.45	16.67	56.25	36.36	64.44	50.00	11.11	**90.57**
Late-Fusion	76.19	54.84	50.00	18.18	46.67	**37.50**	74.42	**72.73**	11.11	86.79
MAG-BERT	63.64	51.79	45.00	0.00	62.50	36.36	64.71	44.44	**16.00**	79.17
MulT	72	51.80	47.83	0.00	45.45	26.67	**76.19**	47.62	14.29	88.46
Ours	64.00	**62.07**	**59.09**	**47.06**	**64.71**	33.33	69.77	64.52	12.50	83.33

Bold text indicates the highest score for each model’s corresponding metric.

## Data Availability

The data presented in this study are available in the public domain. The dataset used in this study is MIntRec (https://github.com/thuiar/MIntRec) (accessed on 10 May 2025). The newly generated data supporting the findings of this study are available from the corresponding author upon reasonable request.
